# The synthesis of poly(vinyl chloride) nanocomposite films containing ZrO_2_ nanoparticles modified with vitamin B_1_ with the aim of improving the mechanical, thermal and optical properties

**DOI:** 10.1080/15685551.2016.1273436

**Published:** 2017-12-28

**Authors:** Shadpour Mallakpour, Elaheh Shafiee

**Affiliations:** ^a^ Organic Polymer Chemistry Research Laboratory, Department of Chemistry, Isfahan University of Technology, Isfahan, Islamic Republic of Iran; ^b^ Nanotechnology and Advanced Materials Institute, Isfahan University of Technology, Isfahan, Islamic Republic of Iran

**Keywords:** ZrO_2_ nanoparticle, surface modification, poly(vinyl chloride), nanocomposites, solution casting method

## Abstract

In the present investigation, solution casting method was used for the preparation of nanocomposite (NC) films. At first, the surface of ZrO_2_ nanoparticles (NPs) was modified with vitamin B_1_ (VB_1_) as a bioactive coupling agent to achieve a better dispersion and compatibility of NPs within the poly(vinyl chloride) (PVC) matrix. The grafting of modifier on the surface of ZrO_2_ was confirmed by Fourier transform infrared spectroscopy and thermogravimetric analysis (TGA). Finally, the resulting modified ZrO_2_ (ZrO_2_–VB_1_), was used as a nano-filler and incorporated into the PVC matrix to improve its mechanical and thermal properties. These processes were carried out under ultrasonic irradiation conditions, which is an economical and eco-friendly method. The effect of ZrO_2_–VB_1_ on the properties and morphology of the PVC matrix was characterized by various techniques. Field emission scanning electron microscopy and transmission electron microscopy analyses showed a good dispersion of fillers into the PVC matrix with the average diameter of 37–40 nm. UV–Vis spectroscopy was used to study optical behavior of the obtained NC films. TGA analysis has conﬁrmed the presence of about 7 wt% VB_1_ on the surface of ZrO_2_. Also, the data indicated that the thermal and mechanical properties of the NC films were enhanced.

## 1. Introduction

The way to expand the nanocomposites (NCs) area is to add modified nanoparticles (NPs) into the polymer matrices or by linkage synthetic polymers onto surface of inorganic particles [1]. NCs obtained by incorporation of different types of nano-fillers such as metal oxide NPs are characterized by improved mechanical, thermal, optical and catalytic properties. NCs are used in a wide range of applications due to their new physical and chemical properties [2].

Excellent chemical resistance, easy modification, nonﬂammability and low production cost of poly(vinyl chloride) (PVC) [3] make PVC-based materials suitable for use as the matrix in the NCs [4]. PVC has been widely used as membrane separation [5], electric cables, clothing and furniture, healthcare and flooring due to its good properties [6, 7]. However, because of its poor processability, low thermal stability [2], nonbiodegradable in normal environment and brittleness has greatly restricted its application [8]. Therefore, it is intransitive to develop new PVC products with reclaim properties [9]. Inorganic nano-fillers are incorporated into the polymer materials to improve their characteristics to make them a suitable material for particular commercial applications. Among all the inorganic nano-fillers, metal oxides such as ZrO_2_ NPs has a great deal of attention, because of their properties such as high thermal stability, high oxygen ion conductivity, high refractive index and band gap [2], mechanical stability and biocompatibility [10].

ZrO_2_ NPs can be directly incorporated into the polymeric matrixes, but because of NPs aggregation which resulted by incompatibility of them within the organic polymers and their specific surface area, it is arduous to produce homogeneous dispersion into the polymer matrix [1, 11].

To get better dispersion stability of NPs into the polymer matrixes, surface modifications of ZrO_2_ NPs with suitable compatibilizers is introduced as an efficient strategy to overcome this problem [12]. Various coupling agents can be applied for surface modification, but among them, biosafe modifiers such as carboxylic acids and oleic acid have been used to improve biocompatibility and biodegradability of organic polymers [11, 13].

Vitamin B_1_ (VB_1_) is a colorless, crystalline, bio-safe, low cost and organosulfur biological compound that is soluble in water and practically insoluble in less polar organic solvents. VB_1_ has several functional groups such as hydroxyl and amino groups that can act as ligand to hydroxyl groups on the surface of ZrO_2_ NPs [14,15]. The ability of VB_1_ for chelate to ZrO_2_ NPs makes it a good candidate as modifier for surface modification of ZrO_2_ NPs [2].

In this work, first a bioactive coupling agent (VB_1_) was chosen for the surface modification of ZrO_2_ NPs in order to achieve excellent dispersion and improve interface between ZrO_2_ NPs and PVC matrix. Finally, the modified ZrO_2_ (ZrO_2_–VB_1_) was integrated into the PVC matrix to improve its thermal, morphological, mechanical and optical properties. All the processes have been carried out by an ultrasonic technique. Ultrasonic irradiation has been widely used in medicine, chemistry and preparing NCs for control size distribution, activation of particles and decreasing the aggregation in the polymer matrix [2, 16].

In all steps, products were studied by different methods such as Fourier transform infrared spectroscopy (FT-IR), UV–Vis spectroscopy, X-ray diffraction (XRD), thermogravimetric analysis (TGA), field emission scanning electron microscopy (FE-SEM), transmission electron microscopy (TEM) and contact angle measurement (surface wettability properties). Finally, tensile tests were performed to characterize the NCs mechanical behavior.

## 2. Experimental

### 2.1. Instrumental analysis

The reactions were performed at room temperature by a TOPSONIC ultrasonic liquid processor (Iran) with power of 400 W in wave frequency of 20 kHz. FT-IR spectra of the specimens were recorded on a Jasco-680 spectrophotometer (Japan) at a resolution of 4 cm^−1^ and scanned at wavenumber range of 400–4000 cm^−1^. FT-IR spectra were used to characterize the chemical bonds of the prepared samples in KBr pills for powder materials and spectrograms of NC films were acquired directly. The XRD patterns were used to characterize the crystalline structure of the samples. The XRD patterns were acquired by using a Philips X’Pert MPD X-ray diffractometer (Germany). The scans were obtained using Cu Kα incident beam (*λ* = 1.5418 Å) at current of 100 mA and voltage of 40 kV with 2θ ranging from 10 to 80° and scanning rate of 0.05° min^−1^ for films and powder specimens. The UV–Vis spectra of the NC films were measured by UV–Vis–Near IR spectrophotometer JASCO, V-570, with solid samples of NC films in the spectral range between 200 and 800 nm. The dispersion morphology of modified NPs and NC films were studied by FE-SEM analysis (Hitachi S-4160, Japan) and TEM images by a Philips CM 120 microscope operated (Netherlands) at voltage of 100 kV. TGA data were done with a STA503 system of TA instrument (Germany) with 20 °C/min rate of heating under argon atmosphere. The temperature was varied from room temperature to 800 °C. In order to test films mechanically, tensile testing was carried out with the speed of 5 mm/min on a Hounsfield test equipment H25KS (RH1 5DZ, England) with a load cell of 60 N at room temperature. The dimensions of the test samples were 35 mm × 9 mm. Contact angle measurement were done with a U-VISION MV500 digital camera microscope (China) .

### 2.2. Materials

Nanosized ZrO_2_ powder was purchased from Neutrino Co. (Iran) with average particle sizes of < 50 nm. Vitamin B_1_ (Mw: 300.81 g/mol) was supplied from Alfa Aesar Chem Co. PVC (Mw: 78,000 g/mol) and THF (Mw: 72.11 g/mol) were obtained from LG Chem Co. (Korea origin) and JEONG Wang Co. (Korea).

### 2.3. Surface modification of ZrO_2_ NPs

**Scheme 1.**
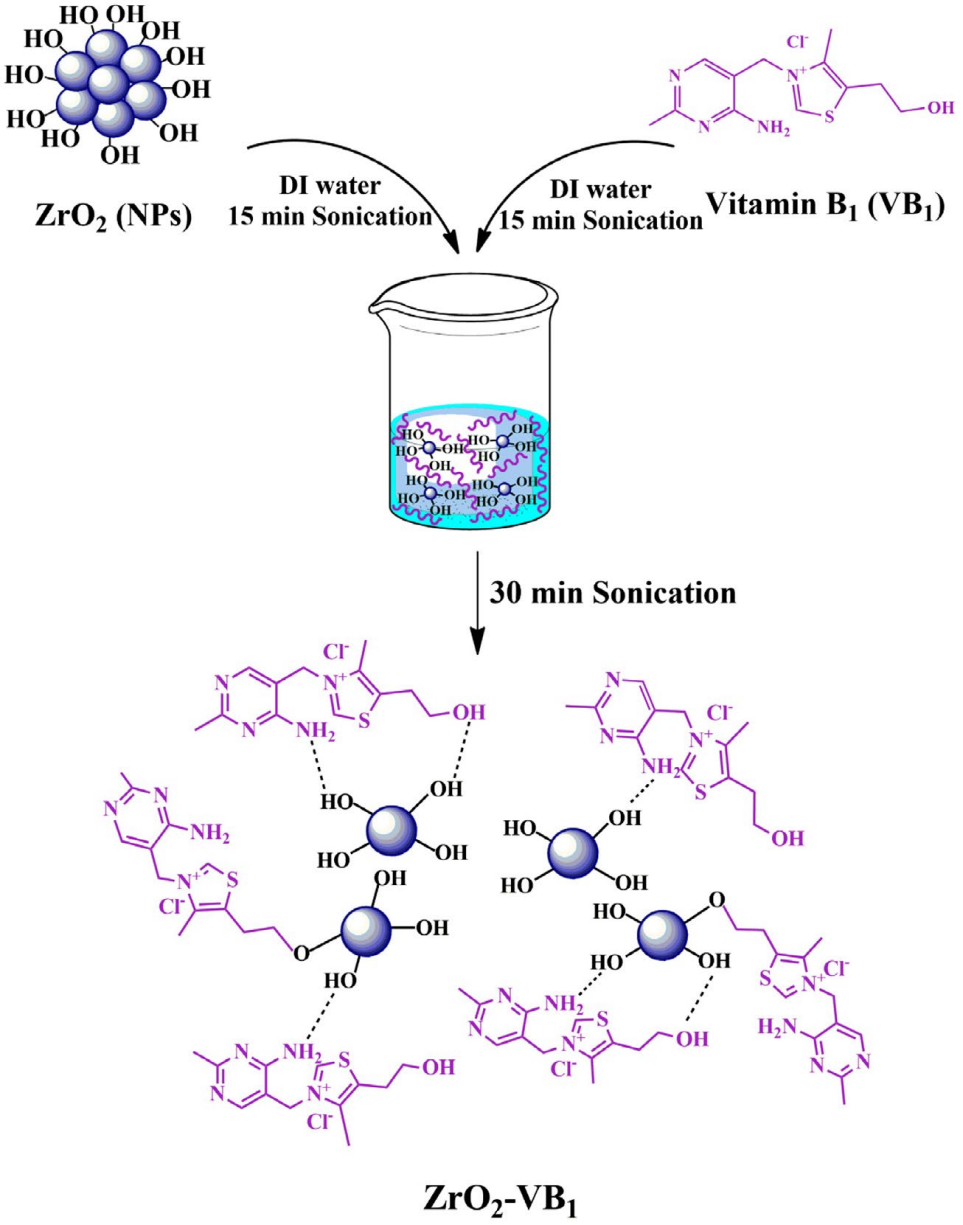
The reaction sequence for surface modification of ZrO_2_ NPs with VB_1_.

For better homogeneous distribution of ZrO_2_ NPs in the PVC matrix, the surface modification of ZrO_2_ NPs must be performed. For the modification of ZrO_2_ NPs with VB_1_: 0.01 g of VB_1_ was added into 8 mL of deionized (DI) water and was ultrasonicated for 15 min. Next, 0.1 g of ZrO_2_ NPs was dispersed in 8 mL of DI water by sonication for 15 min to obtain a suspension of ZrO_2_ NPs. Then, the VB_1_ solution was added to the ZrO_2_ suspension and was sonicated for 30 min under ultrasonic radiations to obtain stable suspension. After irradiation, the mixture was centrifuged, washed thoroughly with water, and dried at room temperature to give the product of ZrO_2_–VB_1_ NPs. The reaction sequence for the functionalization of ZrO_2_ NPs with VB_1_ is shown in Scheme 1.

### 2.4. Fabrication of PVC/ZrO_2_–VB_1_ NC films

**Scheme 2.**
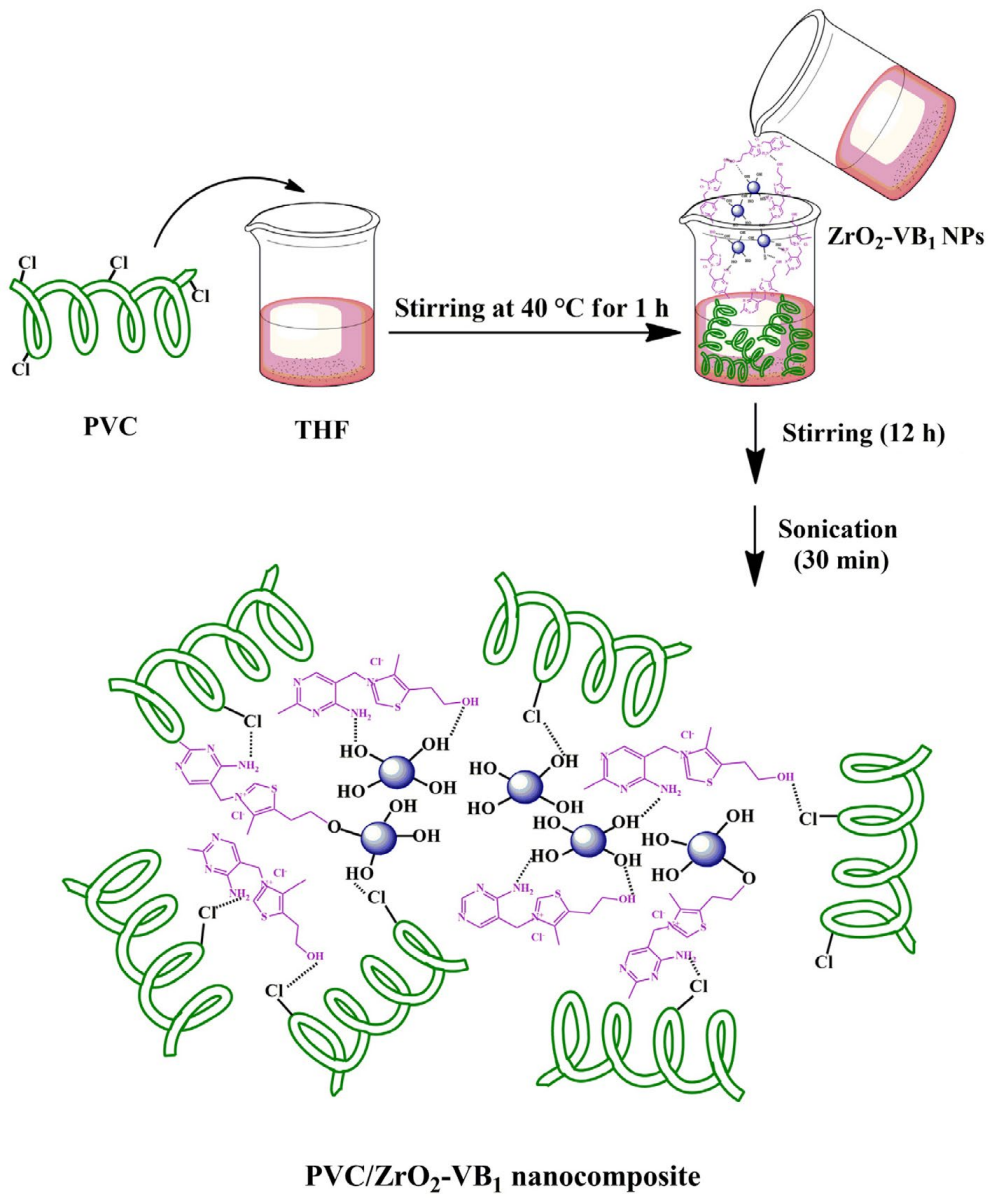
NC structure and some possible interactions between modified NPs and PVC chains.

The PVC/ZrO_2_–VB_1_ NC films were prepared via solution casting, using ultrasonic irradiation method. In this procedure, 0.1 g of PVC was first dissolved in 5 ml of THF and stirred using a magnetic stirrer at 40 °C for 1 h to achieve a homogenous solution. Then, ZrO_2_–VB_1_ NPs with different contents (3, 5 and 7 wt%) were added to PVC solution and stirred for 12 h at room temperature. Finally, the obtained mixtures were ultrasonicated for 30 min and the resulting homogeneous mixtures were casted onto glass Petri dishes and dried at room temperature to prepare NC films. All of these stages were shown in Scheme 2.

**Figure 1.**
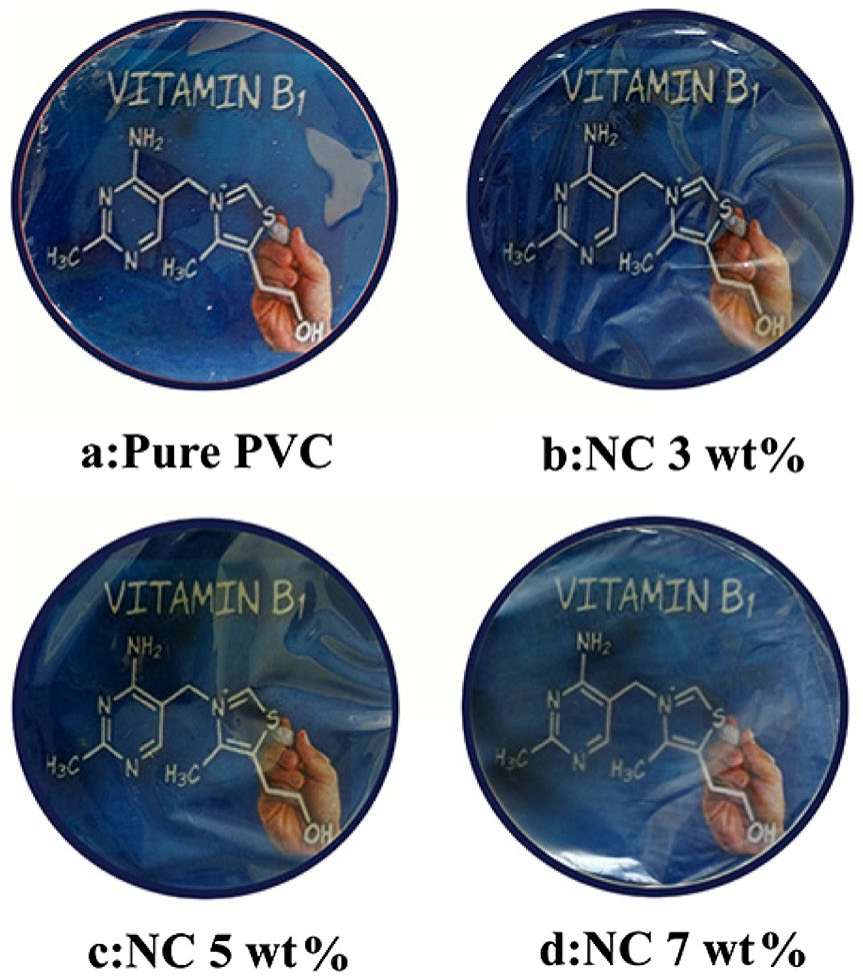
Photographs of (a) pure PVC, (b) PVC/ZrO_2_–VB_1_ NC 3 wt%, (c) PVC/ZrO_2_–VB_1_ NC 5 wt% and (d) PVC/ZrO_2_–VB_1_ NC 7 wt%.

The photographs of the prepared NC films with different contents of ZrO_2_–VB_1_ NPs are shown in Figure 1.

## 3. Results and discussion

### 3.1. Characterization of PVC/ZrO_2_–VB_1_ NC films

ZrO_2_ NP is one of the most interested inorganic nano-filler that exhibits advantages such as UV filtering and thermal stability when inserted in a polymer matrix [17]. Thus, it is extensively used in high-tech applications. Presence of hydroxyl groups on the surface of ZrO_2_ NPs and high surface area cause zirconia aggregates [18]. Surface modification of ZrO_2_ NPs is an efficient strategy to overcome this problem [4]. Because of bioactivity and low cost of VB_1_, it was used as a modifying agent. Due to the presence of numerous hydroxyl and amino groups in the VB_1_, it generates more functional groups and active sites on the ZrO_2_ NP surface. Finally, ZrO_2_–VB_1_ NPs are used for the synthesis of PVC/ZrO_2_–VB_1_ NC films. In fact, the driving force for the adsorption of ZrO_2_–VB_1_ onto the PVC surface is hydrogen bonding. Also, Van der Waals forces between ZrO_2_–VB_1_ NPs surface and PVC segments can be responsible for the overall adsorption process [4]. Ultrasonic irradiation can help better dispersion of NPs in the PVC matrix [16].

### 3.2. FT-IR spectroscopy

**Figure 2.**
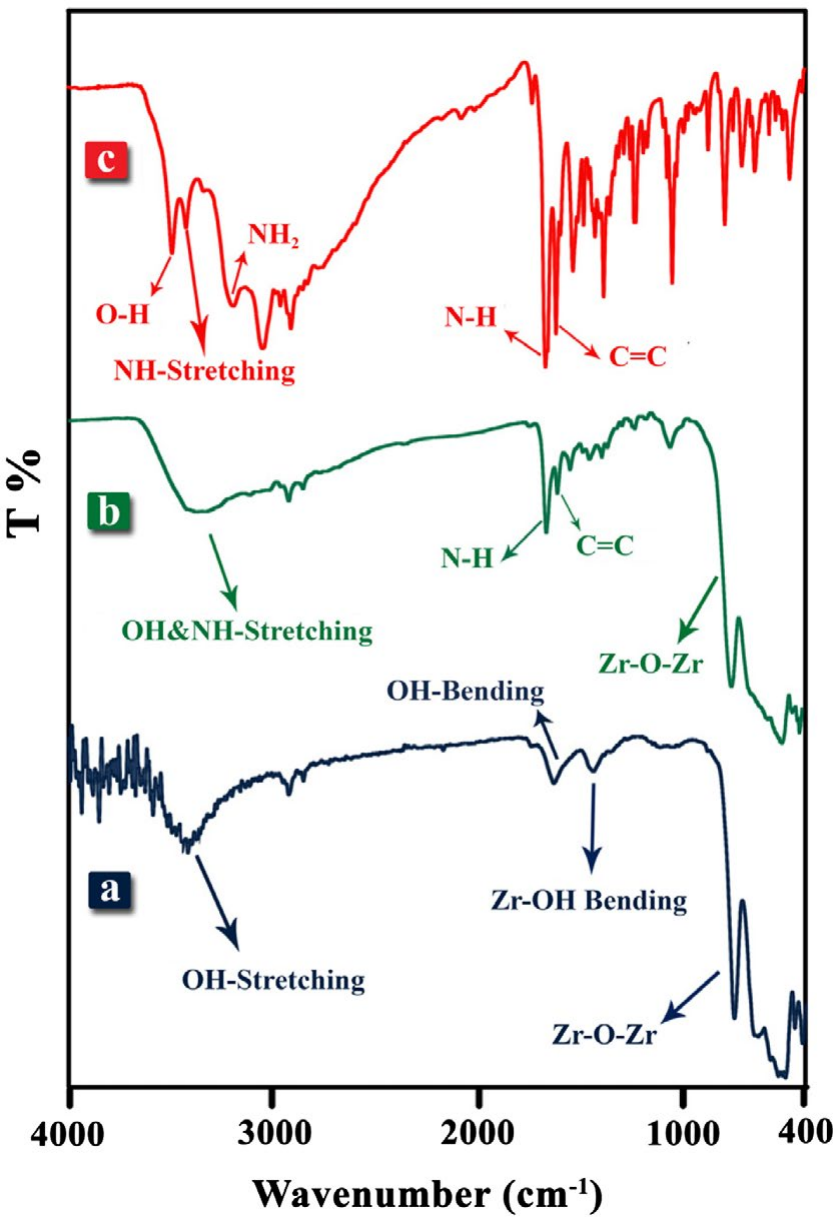
FT-IR spectra of (a) ZrO_2_ NPs, (b) ZrO_2_–VB_1_, (C) VB_1_.

FT-IR spectra of pure ZrO_2_, ZrO_2_–VB_1_ NPs and VB_1_ as coupling agent are shown in Figure 2. In the FT-IR of pure ZrO_2_, the absorption bands at 3448 and 1630 cm^−1^ were corresponded to the stretching and bending vibrations of hydroxyl groups bands, which linked to the ZrO_2_ NPs [19]. The band around about 750 cm^−1^ was attributed to the Zr–O stretching vibration and a band at 1435 cm^−1^ was correspond to the bending vibration of Zr–OH [20]. In the FT-IR spectrum of VB_1_ (Figure 2(c)) a broad band in the region of 2500–3500 cm^−1^ was corresponded to the powerful inter- and intramolecular hydrogen bonding in the VB_1_ [16]. The bands at 3423 and 3185 cm^−1^ were assigned to the –NH and –NH_2_ stretching bands and the band at 3493 cm^−1^ is related to O–H stretching vibration band. The band at 1667 cm^−1^ was attributed to bending of NH (NH_2_). Also, two bands around 1595 and 1615 cm^−1^ were assigned to the C=N and C=C stretching vibration bands in the pyrimidine ring for VB_1_ [15, 21]. The FT-IR spectrum of ZrO_2_–VB_1_ NPs is shown in Figure 2(b). The new peak at 1658 cm^−1^ can be assigned to the NH group in VB_1_. It shows that NH vibration of VB_1_ near 1667 cm^−1^ is shifted to 1658 cm^−1^ in ZrO_2_–VB_1_. The reason of this observation is the existence of interaction between ZrO_2_ NPs and VB_1_. Furthermore, the broad band in region of 2800–3400 cm^−1^ can be related to different hydroxyl and amino groups of VB_1_ [4]. All of these evidences can confirm that the coupling agents have been successfully grafted to the ZrO_2_ NPs surface.

**Figure 3.**
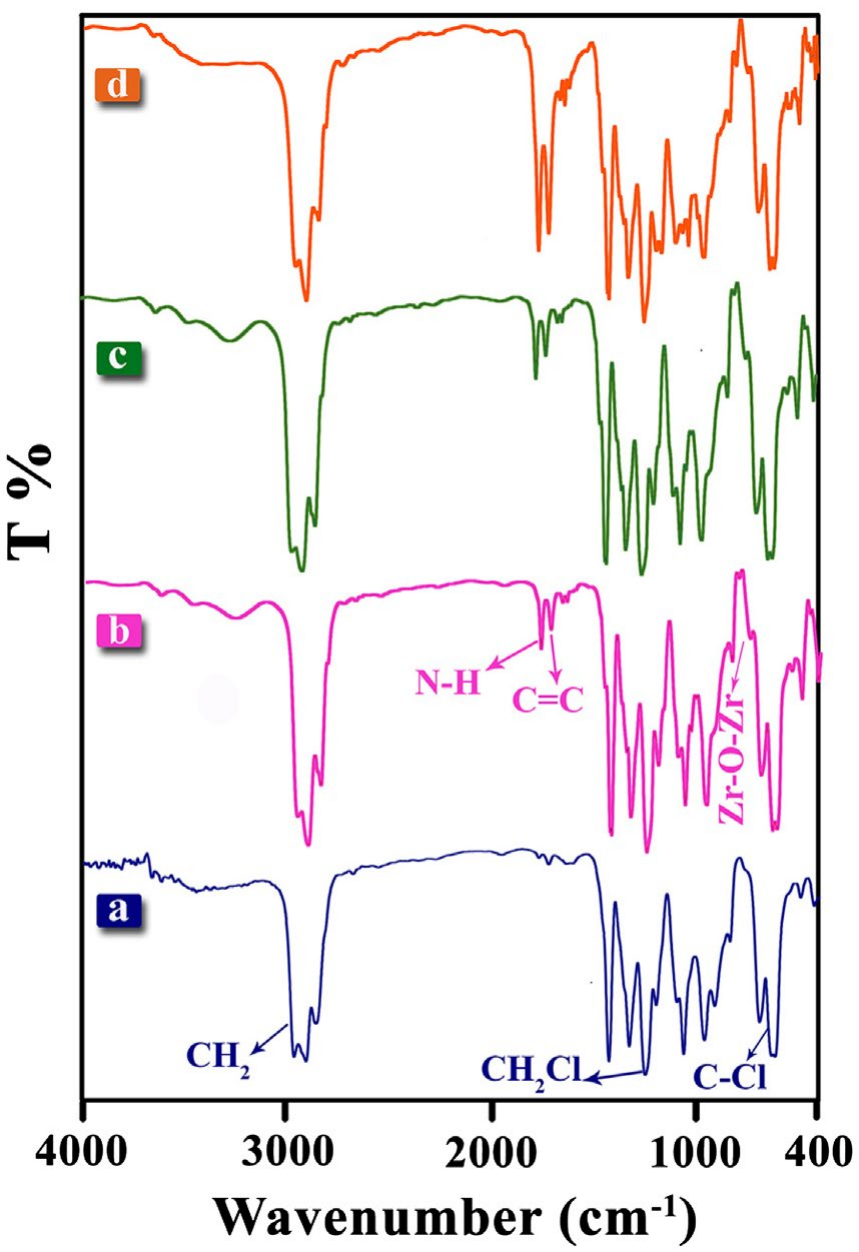
FT-IR spectra of (a) Pure PVC, (b) PVC/ZrO_2_–VB_1_ NC 3 wt%, (c) PVC/ZrO_2_–VB_1_ NC 5 wt% and (d) PVC/ZrO_2_–VB_1_ NC 7 wt%.

Figure 3 illustrates the FT-IR spectra of pure PVC and PVC/ZrO_2_–VB_1_ NC films. In the PVC spectrum, the absorption bands at 616 and 692 cm^−1^ were attributed to the stretching of C–Cl groups [2], The peaks at 1097, 2912, 1252 [3] and 2971 cm^−1^ were related to the C–C, C–H, CH_2_–Cl and CH_2_ stretching vibrations, respectively. PVC/ZrO_2_–VB_1_ NC films (Figure 3(b)–(d)) showed the same spectra, while the band located at 745 cm^−1^ was attributed to the stretching of Zr–O–Zr bond [2]. In addition, the new bands located at 3270, 1667 and 1615 cm^−1^ for NCs were related to N–H and O–H (stretching H-bonded), N–H (bending) and C=C groups of the modifiers that its intensity has been increased with increasing the modified filler content.

### 3.3. Surface morphology analysis

**Figure 4.**
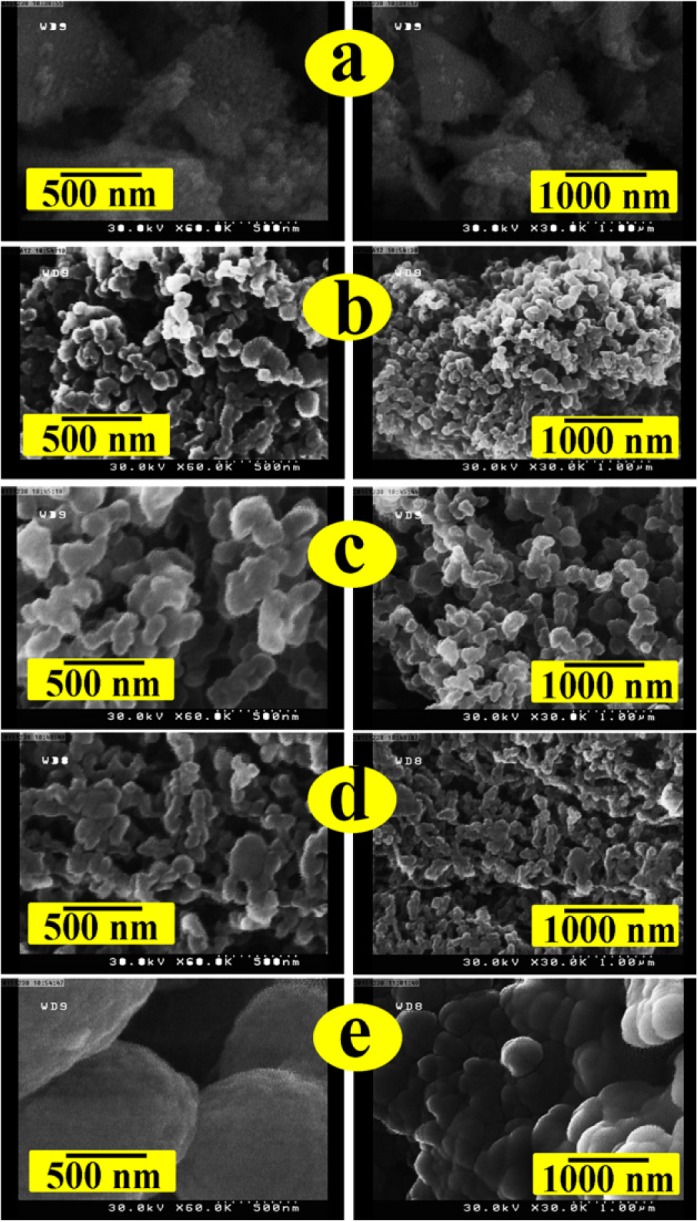
FE-SEM images of (a) ZrO_2_–VB_1_, (b) Pure PVC, (c) PVC/ZrO_2_–VB_1_ NC 3 wt%, (d) PVC/ ZrO_2_–VB_1_ NC 5 wt% and (e) PVC/ZrO_2_–VB_1_ NC 7 wt%.

A high homogenous dispersion of the PVC/ZrO_2_–VB_1_ NC films was fabricated by the interactions of –Cl groups in the PVC chains with –NH and –OH groups on the ZrO_2_–VB_1_ NPs. Figure 4 shows the FE-SEM images of the ZrO_2_–VB_1_, pure PVC and PVC/ZrO_2_–VB_1_ NC films with two different magniﬁcations. It is evident that NPs have homogenous shape. FE-SEM image of the PVC/ZrO_2_–VB_1_ NC films shows that the obtained macromolecules have nanostructures morphology. This phenomenon can be due to the using of ultrasonic technique [22]. According to the obtained results, the morphology of the pure PVC was not changed after incorporation of ZrO_2_–VB_1_ NPs.

**Figure 5.**
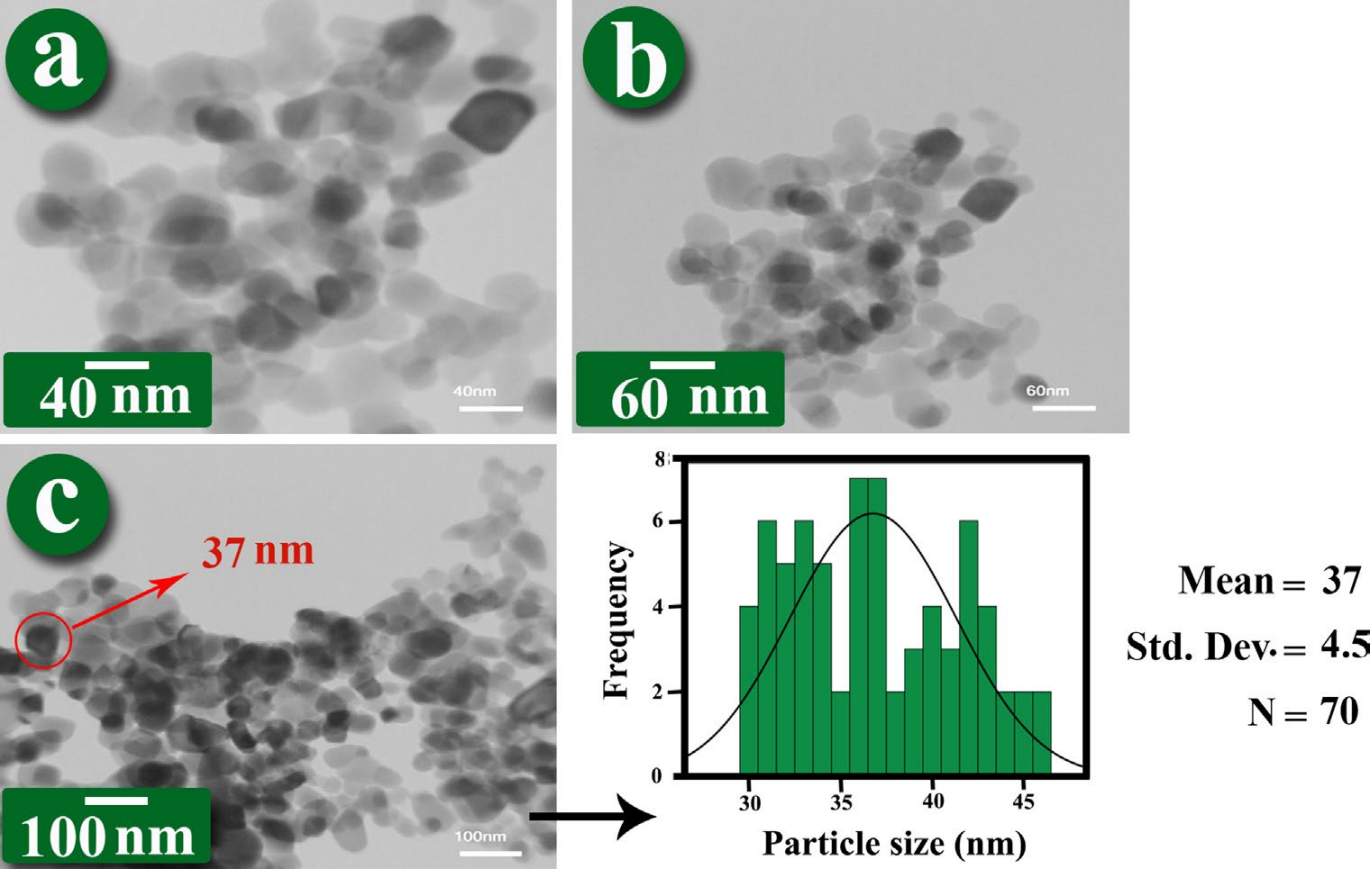
TEM micrographs of ZrO_2_–VB_1_ at different magniﬁcations and its histogram.

**Figure 6.**
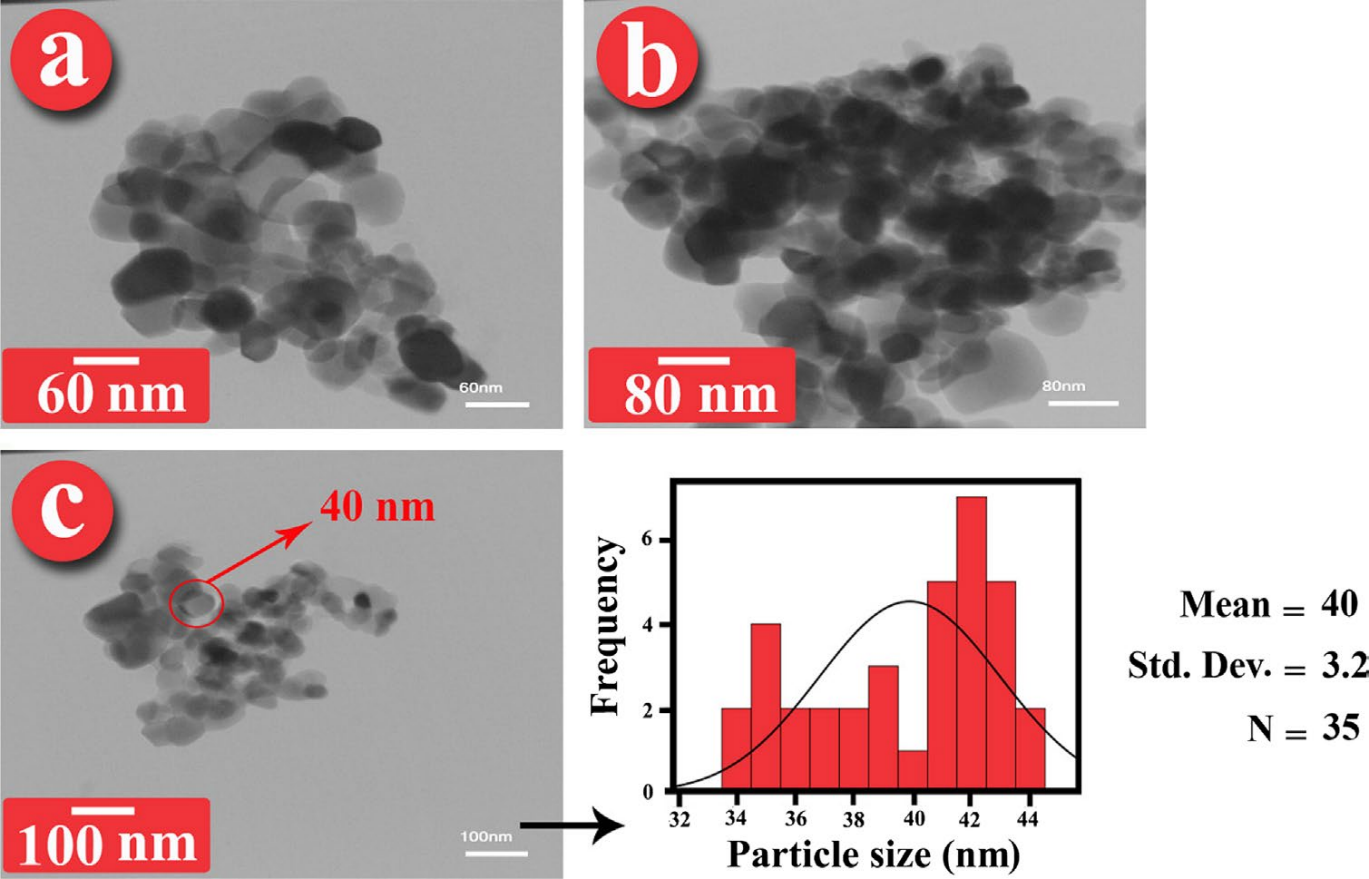
TEM micrographs of PVC/ZrO_2_–VB_1_ NC ﬁlm 3 wt% at different magniﬁcations and its histogram.

These observations were also conﬁrmed by TEM images. Figure 5 shows the TEM images and histogram of ZrO_2_–VB_1_, where the average size of NPs was around 37 nm, as calculated by Digimizer software and SPSS statistics. The images clearly demonstrated that the ZrO_2_–VB_1_ were dispersed in the water and did not show serious aggregation. Figure 6 shows the TEM images and histogram of the PVC/ZrO_2_–VB_1_ NC 3 wt% at different magnifications. From the micrograph, it is observed that the modified NPs has uniform size distribution with an average size of 40 nm in diameter, almost spherical in shape and are well dispersed within the PVC matrix. This result is presumably due to the fact that the surface of ZrO_2_ with high surface energy was modiﬁed with VB_1_, which improved the compatibility of the ZrO_2_–VB_1_ with the matrix.

### 3.4. UV–Vis spectroscopy

**Figure 7.**
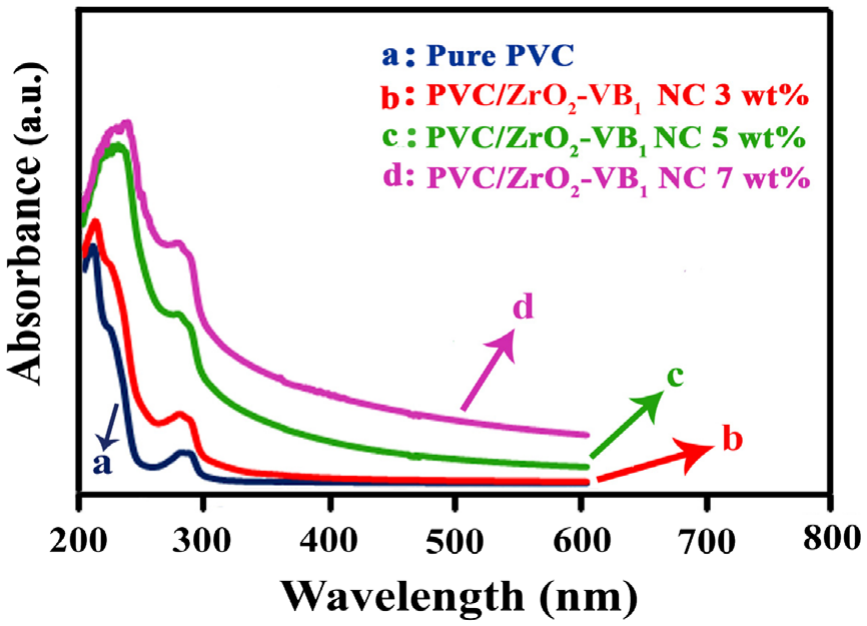
UV–Vis absorption spectra of (a) Pure PVC, (b) PVC/ZrO_2_–VB_1_ NC 3 wt%, (c) PVC/ZrO_2_–VB_1_ NC 5 wt% and (d) PVC/ZrO_2_–VB_1_ NC 7 wt%.

UV–Vis spectra of pure PVC and the NC films with different amounts of ZrO_2_–VB_1_ are shown in Figure 7. It is accepted that the ZrO_2_ is direct band gap insulator, photocatalyst, an active and typical photon absorber. The monoclinic crystalline structure of ZrO_2_ has two direct interband transitions at 5.93 and 5.17 eV. The pure ZrO_2_ NPs have a sharp and intense band at 212 nm with an absorption edge around 300 nm [23, 24]. For the pure PVC, the absorbance spectrum showed absorbance peaks at *λ* = 210 and 280 nm, which can be assigned to the π–π* and n–π* transition, respectively. The significant increase in the absorbance below 256 nm is associated with the C–Cl bond [25, 26]. As shown in Figure 7, the maximum UV absorption of NC films was more than the pure PVC and their absorptions were transferred to higher wavelengths in the visible region. These can be attributed to the existence of the ZrO_2_–VB_1_ NPs, which can increase UV absorption of the NCs because of transfers and conjugate structure of modifier and UV-resistant property of ZrO_2_ NPs. Therefore, the prepared NCs can be used as a UV shielding material.

### 3.5. Thermal resistance

**Figure 8.**
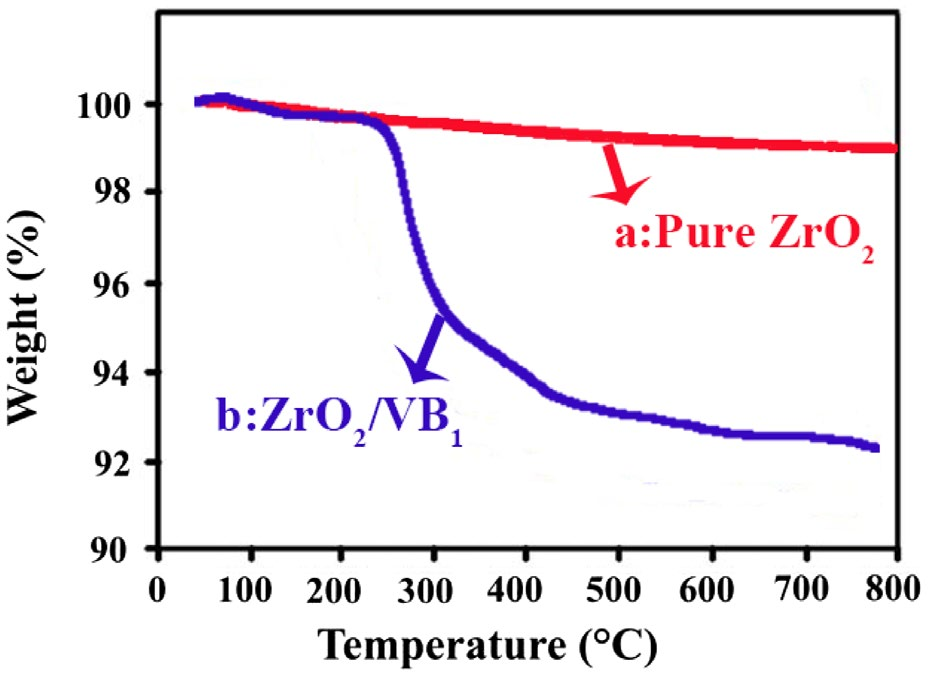
TGA thermograms of (a) Pure ZrO_2_, (b) ZrO_2_–VB_1_.

TGA analysis is an analytical technique used to determine a material’s thermal stability and its fraction of volatile components by monitoring the weight change that occurs as a sample is heated [27]. Figure 8 demonstrates weight loss vs. temperature curve of the pure ZrO_2_ and ZrO_2_–VB_1_ NPs. In decomposition processes, the weight loss below 120 °C was ascribed to physically adsorbed water [3]. In thermogram of ZrO_2_–VB_1_ one step degradation can be seen at around 250 °C, which it may be ascribed to the separating of modifier from the surface of ZrO_2_ NPs. The amount of coated modifiers on the surface of ZrO_2_ NPs can be estimated by comparing the weight loss of pure ZrO_2_ NPs and modified ZrO_2_ NPs from the residue via TGA thermogram. Residual weight loss values at 800 °C for the pure ZrO_2_ NPs is 2 wt% due to the removal of physically adsorbed water and it is 9 wt% for modified ZrO_2_. So, the actual VB_1_ weight percentages supported on the NPs can be calculated and it is about 7 wt%.

As shown in graph, it can be readily predicted that the weight ratio of the grafted VB_1_ on ZrO_2_ surface is approximately around 7 wt%. So, it can be concluded that the modifier was successfully grafted on the surface of NPs.

**Figure 9.**
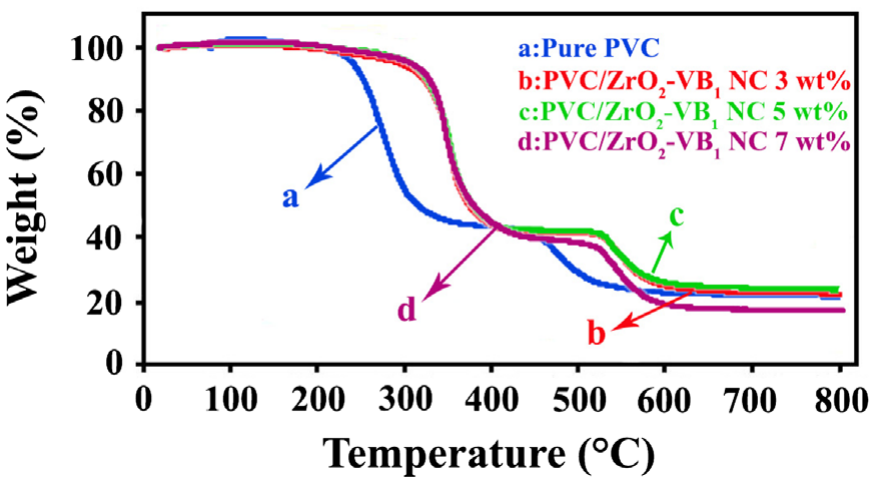
TGA thermograms of (a) Pure PVC, (b) PVC/ZrO_2_–VB_1_ NC 3 wt%, (c) PVC/ZrO_2_–VB_1_ NC 5 wt% and (d) PVC/ZrO_2_–VB_1_ NC 7 wt%.

TGA curves for the pure PVC and PVC/ZrO_2_–VB_1_ NC films of different wt% are shown in Figure 9. The thermal degradation of pure PVC takes place in three mass loss stages. The first stage takes place around 230–350 °C [28], which may be due to the emission of hydrogen chloride (dehydrochlorination), second stage is owing to the thermal cracking of organic materials bonds [4] and the third one in higher temperature region is related to crosslinking (Figure 9(a)). For all NC films, thermal decompositions occurred at higher temperatures than pure PVC.

The temperature at which 5% (T_5_) and 10% (T_10_) degradation occurred, char yield (residues of PVC/ZrO_2_–VB_1_ NC films at 800 °C) and the limiting oxygen index (LOI) based on Van Krevelen and Hoftyzer equation (LOI = 17.5‏ + 0.4 *CY*, where *CY* = char) [2] of the pure PVC and its NCs are calculated and summarized in Table 1. From these data, it can be concluded that NCs containing ZrO_2_ NPs have a higher decomposition temperature in contrast to the pure PVC. In fact, the presence of the ZrO_2_–VB_1_ could restrict the mobility and ﬂexibility of the PVC chains and lead to an increase in the decomposition temperature [4]. Char yield values were increased with increasing the amount of ZrO_2_–VB_1_ except in the case of 7 wt% that may be due to the partial aggregation and reduce the effect of NPs on the polymer. It is reported that materials with LOI values more than 21 can show self-extinguishing behavior and considerate as flame retardants, so these NCs can be classified as the self-extinguishing materials [3].

**Table 1.** TGA data for the PVC and prepared NC ﬁlms.

Samples	T_5_^a^	T_10_^b^	Char yield^c^ (%)	LOI^d^
Pure PVC	240	254	23.32	26.82
PVC/ZrO_2_–VB_1_ NC 3 wt%	300	324	24.68	27.37
PVC/ZrO_2_–VB_1_ NC 5 wt%	312	330	25.37	27.64
PVC/ZrO_2_–VB_1_ NC 7 wt%	311	332	19.66	25.36

^a^ Temperature at which 5% weight loss was verified by TGA.

^b^ Temperature at which 10% weight loss was recorded by TGA.

^c^ Percentage weight of material left undecomposed after TGA analysis at maximum temperature 800 °C in an argon atmosphere.

^d^ Limiting oxygen index (LOI) calculated at char yield at 800 °C.

### 3.6. XRD analysis

**Figure 10.**
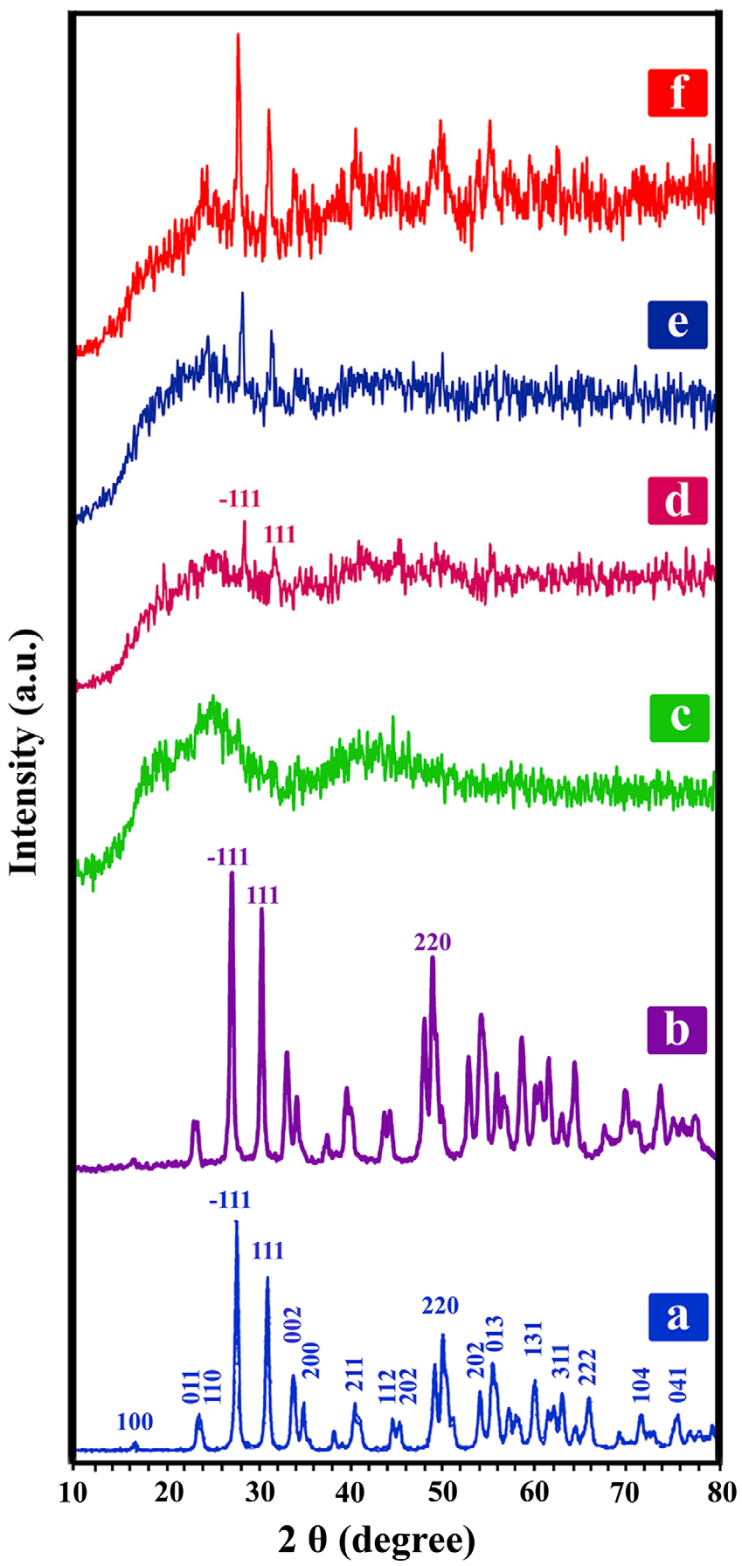
XRD spectra of (a) ZrO_2_ NPs, (b) ZrO_2_–VB_1_, (c) Pure PVC, (d) PVC/ZrO_2_–VB_1_ NC 3 wt%, (e) PVC/ZrO_2_–VB_1_ NC 5 wt% and (f) PVC/ZrO_2_–VB_1_ NC 7 wt%.

X-ray diffraction measurements were conducted to examine the nature of crystallinity of the NC films with respect to pure PVC film and to investigate the occurrence of complexation between the polymer matrix and the modified NPs [27]. The XRD patterns of pure ZrO_2_, ZrO_2_–VB_1_, pure PVC and related NCs with different ZrO_2_–VB_1_ percentages are shown in Figure 10. The characteristic peaks of ZrO_2_ NPs are distinctly observed at 2*θ* = 28°, 32°, 36°, 47°, 56°, 59°, 68°, 76° and 79° [29]. These characteristic peaks corresponded to highly crystalline monoclinic structure of single-phase ZrO_2_ [2, 23]. ZrO_2_–VB_1_ NPs pattern (Figure 10(b)) exhibits a series of characteristic peaks that are as same as pure ZrO_2_ peaks, which may be due to the low amounts of VB_1_. So, there is no change in the crystalline structure of ZrO_2_ after the surface modification. PVC is an amorphous thermoplastic, which has no regular crystalline planes with high intensity peaks (Figure 10(c)) [4]. The XRD of the NCs (Figure 10(d)–(f)) displays two main monoclinic diffraction lines which are related to the crystals planes (−111) and (111) as well as broad noncrystalline peak of the PVC [12]. The stronger diffraction intensity of PVC/ZrO_2_–VB_1_ NC 7 wt% is attributed to the increase in crystallinity fraction due to the incorporation of ZrO_2_–VB_1_ within the PVC NC films.

### 3.7. Water contact angle and wetting properties

**Figure 11.**
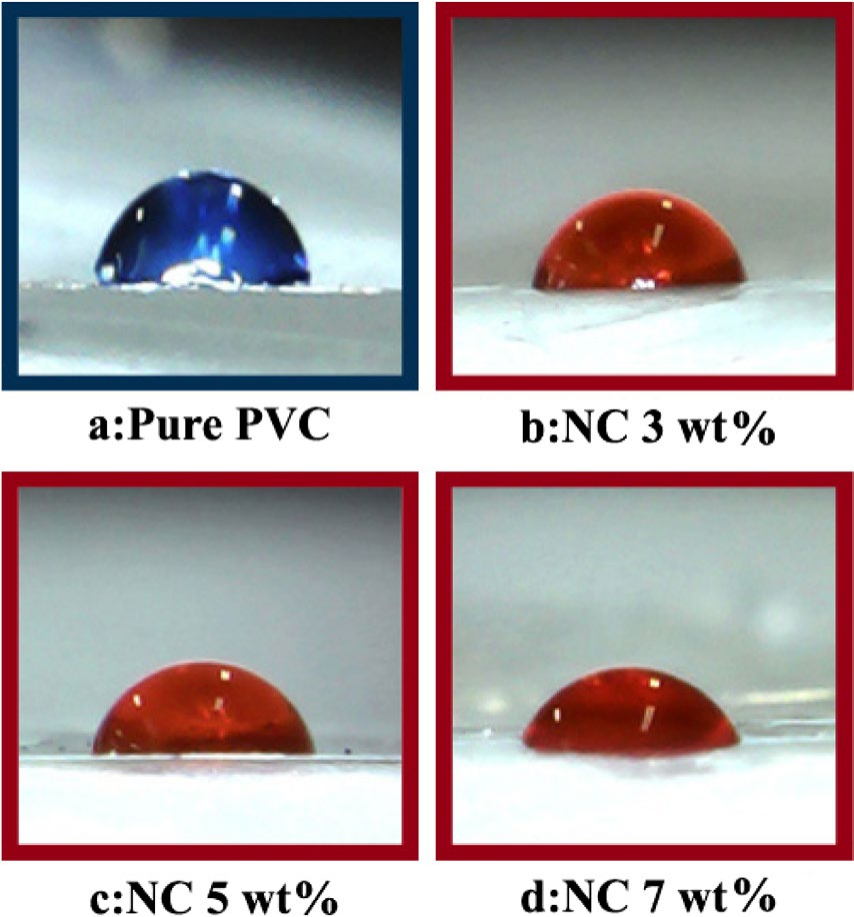
Pictures of water droplets on (a) Pure PVC, (b) PVC/ZrO_2_–VB_1_ NC 3 wt%, (c) PVC/ZrO_2_–VB_1_ NC 5 wt% and (d) PVC/ZrO_2_–VB_1_ NC 7 wt%.

A water contact angle method is used to characterize the surface properties by measuring how much a water droplet could spread on a surface [30]. Figure 11 shows pictures of water droplets on the pure PVC and PVC/ZrO_2_–VB_1_ NC films. Measurements were made at 3 different locations on the sample and the water contact angles at the 3 different locations on the surface were averaged. In this study, we observed decreases in the contact angle values of PVC/ZrO_2_–VB_1_ NC films with increasing the ZrO_2_–VB_1_ content. Contact angles of water droplets were measured and summarized in Table 2.

**Table 2.** Contact angles of water droplets data for the PVC and prepared NC films.

Samples	Contact angles (°)
Pure PVC	67.12 ± 1.18
PVC/ZrO_2_–VB_1_ NC 3 wt%	63.14 ± 4.66
PVC/ZrO_2_–VB_1_ NC 5 wt%	60.47 ± 9.65
PVC/ZrO_2_–VB_1_ NC 7 wt%	58.87 ± 1.01

Inorganic nano-fillers like ZrO_2_ NPs are of greater surface energy compared to polymer matrix. Therefore, addition of these NPs to hydrophobic materials such as PVC results in an increase in surface free energy and reduction in contact angle [31]. Also, the amount of adsorbed water on the NC films is dependent on the surface density OH and NH groups of ZrO_2_–VB_1_ NPs, which can form hydrogen bonds with water molecules.

### 3.8. Mechanical properties

**Figure 12.**
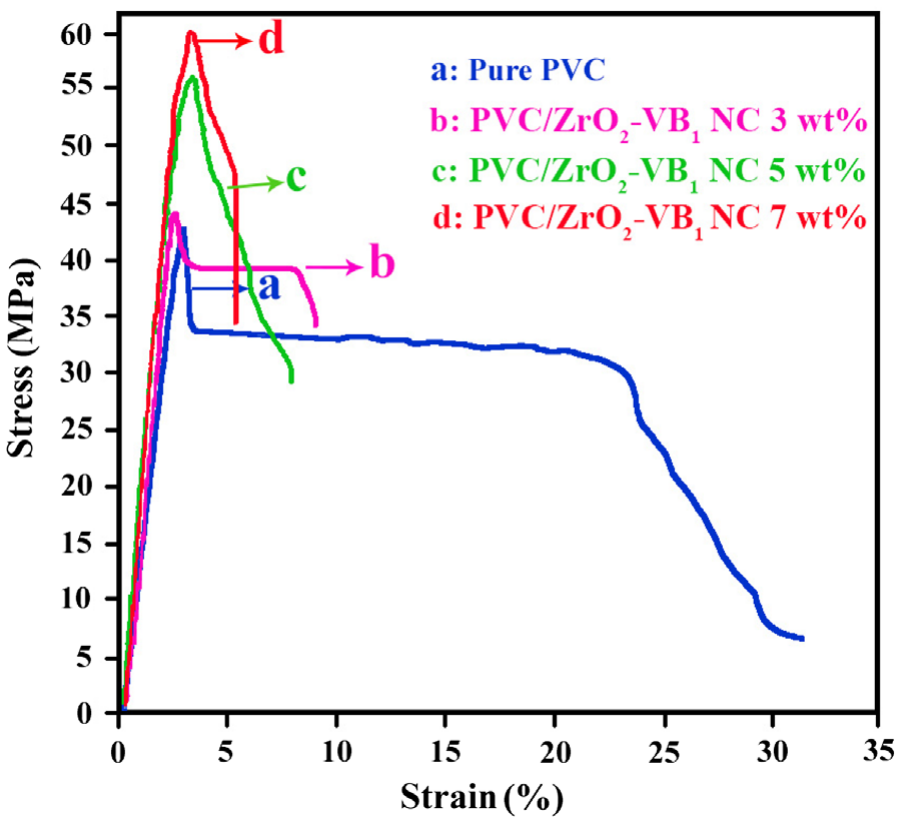
Mechanical properties of (a) Pure PVC, (b) PVC/ZrO_2_–VB_1_ NC 3 wt%, (c) PVC/ZrO_2_–VB_1_ NC 5 wt% and (d) PVC/ZrO_2_–VB_1_ NC 7 wt%.

Figure 12 describes the stress–strain curves of PVC and NC films containing 3, 5 and 7 wt% of ZrO_2_–VB_1_ NPs. The results of the mechanical tests, including the tensile strength, strain, Young’s modulus and the elongation at max are summarized in Table 3. As it can be seen from Table 3, it is important to mention that stress, elongation at max and E-modulus were increased for NCs, however strain decreased compared to the pure PVC. It has been reported that the tensile stress and Yang’s modulus are mainly depend on the filler dispersion and also the interaction of them with polymer matrix [32]. With the insertion of ZrO_2_–VB_1_ filler in to the PVC matrix, the tensile stress of NCs were increased which indicates the good dispersion and well interfacial adhesion (between NPs and polymer matrix), caused superior load transfer from the matrix to the reinforcement. In addition, compared to the pure PVC, the elongations at *F*_max_ for NCs were increased with incorporation of ZrO_2_–VB_1_. The possible reason for this observation may be due to decreasing the intermolecular interactions density in the polymer chains and increasing the free volume between the polymer chains after incorporation of NPs which increase the flexibility of NCs [2].

**Table 3.** Mechanical properties from tensile testing for pure PVC and PVC/ZrO_2_–VB_1_ NCs.

Samples	Stress (MPa)	Strain (%)	Young’s modulus (GPa)	Elongation at Max (%)
Pure PVC	41.33	2.65	2.06	0.93
PVC/ZrO_2_–VB_1_ NC 3 wt%	41.13	2.23	2.00	6.02
PVC/ZrO_2_–VB_1_ NC 5 wt%	55.90	2.46	2.38	7.04
PVC/ZrO_2_–VB_1_ NC 7 wt%	58.70	2.89	2.08	5.06

## 4. Conclusions

In this study, the surface of ZrO_2_ NPs was modified with a biosafe molecule (VB_1_). FT-IR and TGA analyses showed that modifier was grafted onto the surface of NPs. TEM and FE-SEM confirmed that NPs dispersed well after the surface modification. Then, the PVC/ZrO_2_–VB_1_ NC films were prepared by adding different amounts of ZrO_2_–VB_1_ NPs into the PVC matrix through solution casting method under ultrasonic irradiation as a green and an easy tool. The thermal, mechanical, morphology, wettability and optical properties of the PVC/ZrO_2_–VB_1_ NC films were determined. The morphology of the obtained NC films is not different from pure PVC and showed homogeneous dispersion of ZrO_2_–VB_1_ in the PVC matrix. TEM micrographs of the PVC/ZrO_2_–VB_1_ NC films revealed that maximum frequency of particle sizes was in the range of 36–40 nm. The results of thermal properties indicated that thermal stability of the PVC films is enhanced after incorporation of the ZrO_2_–VB_1_ NPs. PVC water absorption has increased by incorporating ZrO_2_–VB_1_. UV–Vis diagrams showed that NC films have more optical absorption than pure PVC. According to the XRD results, the modification process had no effect on ZrO_2_ crystalline structure. Also, mechanical tests indicated that the NC films were more flexible than the pure polymer. Therefore, the surface modification of ZrO_2_ NPs and its loading into the PVC matrix improved the polymer properties.

## Disclosure statement

No potential conflict of interest was reported by the authors.

## Funding

This research was financially supported by the Research Affairs Division Isfahan University of Technology (IUT), Isfahan, Iran.
